# Feasibility of an Emergency Department-based Food Insecurity Screening and Referral Program

**DOI:** 10.5811/westjem.40006

**Published:** 2025-03-15

**Authors:** Victor Cisneros, Ian Dennis Capo Olliffe, Marco Santos Esteban, Joseph Bui, Armin Takallou, Shahram Lotfipour, Bharath Chakravarthy

**Affiliations:** * Eisenhower Health, Department of Emergency Medicine, Rancho Mirage, California; † University of California, Irvine, Department of Emergency Medicine, Irvine, California; ‡ Oregon Health and Sciences University, Portland, Oregon

## Abstract

**Introduction:**

Food insecurity (FI) remains a pervasive issue in the United States, affecting over 12.8% of households. Marginalized populations, particularly those in urban areas, are disproportionately impacted. The emergency department (ED) holds potential as a vital outreach hub, given its diverse patient population and extensive service coverage. In this study we explore the feasibility of implementing an ED-based FI screening and referral program at an urban, academic teaching hospital. We aimed to assess the prevalence of FI among ED patients and evaluate the feasibility of a three- and six-week follow-up to assess patients’ FI and related barriers to resource referral utilization.

**Methods:**

This single-center, observational study was conducted at an urban, academic ED from 2018–2024. Initial FI screening was performed using a validated two-question survey adapted from the Hunger Vital Sign screening tool. Participants who screened positive were enrolled and completed the 10-item US Department of Agriculture Adult Food Security survey, received a food assistance guide, and were followed up at three- and six-week intervals to assess changes in FI status.

**Results:**

Among 6,339 participants, 1,069 (16.9%) experienced FI, with the highest rates among Black non-Hispanic (24.7%) and Spanish-speaking participants (28.7%). Of the 1,069 participants who screened positive for FI, 630 (59.0%) were enrolled in the study. Of the enrolled participants, 161 (25.6%) completed the three-week follow-up phone calls, and 48 (7.6%) completed the six-week follow-up. The mean FI score for these 48 participants decreased from 6.67 (SD 2.68) at enrollment to 4.75 (SD 2.85) at the three-week follow-up (*P* < 0.001), and to 4.25 (SD 3.48) by the six-week follow-up (*P* < 0.001). Barriers to using the food resource guide, such as time constraints, transportation, and misplacement of resources, limited many participants’ engagement.

**Conclusion:**

This study demonstrated the feasibility and effectiveness of an ED-based food insecurity screening and resource referral program, associated with a significant reduction in food insecurity scores among participants. However, barriers such as time constraints, transportation issues, and misplacement of referral materials limited engagement. Addressing these barriers through tailored follow-up and systematic support systems, including universal screening during ED intake and personalized assistance, can enhance the program’s accessibility and impact.

## INTRODUCTION

Food insecurity (FI), defined as limited or uncertain access to adequate food for an active, healthy life, continues to affect over 12.8% of US households, including 44.2 million individuals and 7.3 million children by the end of 2022.^1^ Despite hopes for improvement following the COVID-19 pandemic, ongoing challenges such as inflation and supply chain disruptions have exacerbated vulnerabilities, particularly among marginalized populations.^2^ In Orange County, California, 10.4% of adults faced food insecurity as of 2022, highlighting persistent disparities even in economically prosperous regions.^3^ The consequences of FI extend beyond immediate hardship, with long-term health impacts including chronic illnesses like diabetes and obesity, driven by reliance on cheaper, calorie-dense, and nutritionally inadequate foods.^4^–^6^ Financial strain often forces individuals to choose between food and other essentials such as medications, which leads to worsening health outcomes in people with lower socioeconomic status.^7^

While primary care settings are essential for interventions, the emergency department (ED) holds potential as a vital outreach hub, given its diverse patient population and extensive service coverage. A notable proportion of ED patients in both pediatric and adult hospitals experience FI, with previous rates of FI reported above 20%.^8^ While existing ED-based FI screening and referral programs for FI have primarily focused on screening using tools such as the Hunger Vital Sign,^2^,^9^ few studies have examined the implementation of an ED-based FI screening coupled with a referral program that includes patient follow-ups, assessment of FI severity in food insecure patients, and investigation of barriers to utilization of referral resources. Recognizing this gap, we sought to investigate the feasibility and effectiveness of implementing a FI screening and referral program with patient follow-ups in an urban, academic ED. We hypothesized that implementing a food resource referral program in the ED would significantly reduce FI among patients, as evidenced by lower FI scores in follow-up assessments. We also aimed to investigate the barriers ED-presenting patients have in using FI resources.

## METHODS

### Part 1: Screening

This single-center, observational study was conducted with institutional review board approval at a large, urban, academic medical center ED and adhered to federal guidelines. Research associates (RA) stationed at the ED from 8 am to midnight, May 2018–October 2024, conducted the screening process. Inclusion criteria were as follows: adults ≥18 years of age who were able to understand and communicate in English or Spanish and exhibited full cognitive abilities to provide informed consent. Exclusion criteria included critically ill patients, non-communicative patients, those under <18, psychiatric patients, and those who spoke languages other than English or Spanish.

**Population Health Research Capsule**

What do we already know about this issue?

*ED patients have elevated food insecurity (FI) rates; FI screening is feasible, but the impact of resource referrals and utilization barriers are not well understood.*

What was the research question?

*Can ED-based FI screening and resource referral reduce FI, and what are the barriers to resource utilization?*

What was the major finding of the study?

*FI prevalence in the ED was 16.9%; FI scores decreased from 6.67 to 4.75 at 3 weeks (P < 0.001).*

How does this improve population health?

*ED-based interventions can reduce FI. Identifying and addressing utilization barriers can improve access to essential resources for those most at risk.*

The preliminary assessment of FI was based on a validated and abbreviated two-question survey called the Hunger Vital Sign, which indicates FI adapted from the US Department of Agriculture (USDA) Adult Food Security Survey Module (Supplementary File A).^10^, ^11^ Participants were selected using convenience sampling during the RAs’ working hours and were administered the screening questions orally. For non-English speaking participants, bilingual RAs translated and orally conducted the survey. A positive response to either of the preliminary survey questions classified the individual as experiencing FI, leading to further steps in the enrollment process. Conversely, those who responded negatively had their data anonymized and did not proceed further in the study. This streamlined two-question assessment ensured that targeted assistance and resource guides to food pantries were directed exclusively to individuals identified as food insecure, while assessing the percentage of individuals that were food insecure in the ED in the context of all patients.

### Part 2: Enrollment

After the initial two-question survey, patients identified as food insecure were invited to enroll in the second portion of the study. The research team provided an overview of the study objectives, procedures, potential risks, and benefits, and answered any questions. Written informed consent was obtained prior to participation. After consent, participants completed the 10-item three-stage design USDA Adult Food Security Survey Module to assess the severity of their FI (Supplementary File A).^11^ Additional data collected included demographic information such as age, gender, race/ethnicity, language preference, and other relevant socioeconomic factors. Participants were provided with a food assistance and resource guide, which included information on existing food assistance programs, such as the Supplemental Nutritional Assistance Program/CalFresh, as well as enrollment instructions and contact information for 2-1-1 Orange County.^12^, ^13^ The guide also contained a comprehensive list of local food pantries, sourced from the Second Harvest Food Bank digital resource (Supplementary File B).^14^ The RAs explained the contents of the guide and how to access the resources.[Fig f1-wjem-26-396]

### Part 3: Follow-up

Following enrollment, telephone surveys were scheduled at three- and six-week intervals. During these calls, a modified version of the USDA Adult Food Security Survey Module was administered, adjusted to assess food security status over the prior three weeks instead of the standard 12-month period. The purpose of these calls, conducted by designated RAs, was to assess the impact of FI interventions on improving food accessibility for patients. These calls were not recorded; however, responses were securely stored using Research Electronic Data Capture tools (REDCap) hosted at University of California, Irvine.

#### Sample Size

A university-affiliated statistician conducted power analyses to estimate the required sample size for each survey component. Based on these calculations, we aimed to recruit at least 4,900 participants for the initial two-item screening tool to achieve sufficient power. For the 10-item USDA Adult Food Security Survey Module, an estimated 85 participants were required for adequate power; to account for attrition, we targeted enrolling at least 95 individuals. Due to greater-than-anticipated attrition during follow-up, we continued enrollment beyond initial estimates.

#### Statistics

We calculated descriptive statistics for demographic variables and the prevalence of FI. Differences in FI prevalence across demographic groups, including gender, race/ethnicity, language, and age, were assessed using chi-squared tests. We used paired *t*-tests to compare mean FI scores at enrollment, and at three-week and six-week follow-ups among participants who completed all follow-ups. Independent samples *t*-tests evaluated differences in FI scores between participants who used the food resource guide and those who did not. We conducted multiple linear regression analysis to assess predictors of change in FI scores, adjusting for baseline FI score and potential confounders such as age, gender, ethnicity, language, marital status, and education level. Non-response bias was evaluated by comparing baseline demographics between respondents and non-respondents through logistic regression analysis, following Phillips et al (2015).^15^ Due to study constraints, it was not feasible to perform wave analysis and follow-up analysis of nonresponse bias. We analyzed qualitative data on barriers to resource utilization using inductive coding of interview notes. All statistical analyses were performed using RStudio software (RStudio PBC, Boston, MA), with a significance level set at *P* < 0.05.

## RESULTS

**Figure 2:**
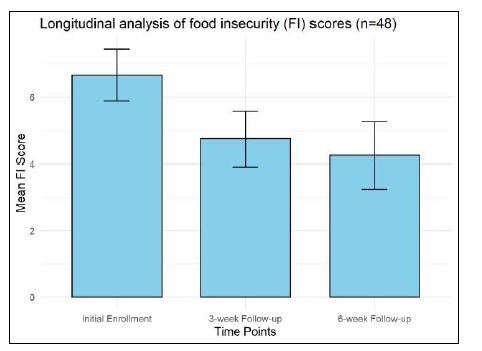
Longitudinal analysis of food insecurity (FI) scores among 48 participants who completed all follow-up surveys. *FI*, food insecurity.

A total of 6,339 participants were screened for FI at a large, urban, academic medical center ED between May 2018–October 2024. The overall prevalence of FI was 16.9% (1,069 of 6,339 participants). When stratified by gender, the FI rate was 17.9% among men and 15.8% among women (*P* = 0.02). Racial and ethnic disparities were evident: Asian or Pacific Islanders and White non-Hispanic individuals had the lowest FI rates at 9.1% and 13.8%, respectively. Black non-Hispanic individuals had the highest at 24.7%. American Indian, Alaskan, or Hawaiian Natives had an FI rate of 23.3%, and Hispanic or Latino/a individuals had an FI rate of 21.6%. Among language groups, Spanish speakers exhibited the highest FI rate at 28.7%, compared to 15.9% for English speakers. Age also influenced FI rates, with the 45–59 age group experiencing the highest rate at 22.6%, and those ≥60 of age the lowest at 11.3%.Of the 1,069 participants who screened positive for FI, 630 (59.0%) were enrolled in the study. Among the enrolled participants, 161 (25.6%) completed the three-week follow-up phone calls, and 48 (7.6%) completed the six-week follow-up. Among the 48 participants who completed all follow-up surveys, the mean FI score decreased from 6.67 (SD 2.68) at enrollment to 4.75 (SD 2.85) at the three-week follow-up (*P* < 0.001), and to 4.25 (SD 3.48) at the six-week follow-up ((*P* < 0.001). The change between the three-week and six-week follow-ups was not statistically significant ((*P* = 0.25), suggesting stabilization of FI scores after the initial intervention (Figure 2). We used paired *t*-tests for these comparisons.[Table t1-wjem-26-396]

We conducted a comparison of FI scores between the 35 participants who used the food resource guide and the 126 who did not. An independent samples *t*-test revealed that guide users had a higher mean initial FI score (7.60 ± 2.43) compared to non-guide users (6.46 ± 2.86; *P* = 0.02). At the three-week follow-up, paired samples *t*-tests showed that FI scores decreased significantly in both groups (guide users: 4.03 ± 3.08, *P* < 0.001; non-guide users: 4.49 ± 3.44, *P* < 0.001). Furthermore, an independent samples *t*-test comparing the unadjusted difference in the decrease in FI scores between the groups was statistically significant (*P* = 0.03). However, this initial observation did not account for baseline differences between the groups.

To account for baseline differences and potential confounders, we conducted a multiple linear regression analysis with FI change (follow-up FI score minus initial FI score) as the dependent variable. The model adjusted for baseline FI score, age, gender, ethnicity, language, marital status, and education level (adjusted R^2^ 0.281, Table 2). The regression analysis revealed that guide use was not a significant predictor of FI change (β -0.649, SE 0.645, t −1.006, *P* = 0.316). This indicates that, after adjusting for other factors, participants who used the guide did not experience a significantly greater reduction in FI scores compared to those who did not use the guide.

Higher baseline FI scores were significantly associated with greater reductions in FI scores (β −0.655, SE 0.095, t −6.907, *P* < 0.01), suggesting that participants with higher initial FI experienced more substantial improvements over time. Ethnicity also emerged as a significant factor. Hispanic or Latino/a participants showed significantly greater reductions in FI scores compared to White non-Hispanic participants (β −1.284, SE 0.615, t −2.087, *P* = 0.039).To evaluate potential non-response bias due to the low response rate during follow-up phone calls, we conducted a logistic regression analysis comparing baseline demographics between those who completed follow-up surveys and those who did not. The analysis identified that participants identifying as Black non-Hispanic (*P* = 0.02) and those with education levels of elementary/high school/General Educational Development (*P* = 0.04), two-year college (AA/AS) (*P* = 0.01), and Master’s degree (*P* = 0.02) were significantly less likely to complete follow-up surveys compared to their respective reference groups. These findings suggest that non-response bias may be present, particularly among Black non-Hispanic participants and individuals with certain educational backgrounds.

**Figure 3:**
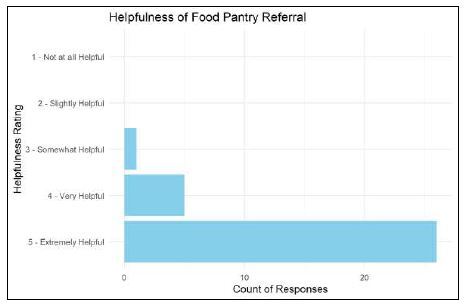
Helpfulness of food resource referral rated by participants during phone follow-ups.

The perceived helpfulness of the food resource referral was assessed during phone call follow-ups with patients who cited usage of the referral. They were asked: “On a scale of 1 to 5, how helpful was this food resource referral in reducing your concerns about food availability?” The majority of participants found the referral to be extremely helpful (Figure 3).

Of the 163 patients who completed the three-week follow-up, 128 did not use the resource guide. Inductive coding revealed that the most frequently reported barriers included time constraints (20%), transportation issues (16%,), and medical concerns (14%). Some participants misplaced or did not recall the referral paper (13%) or simply forgot about it (9%). Other reasons ranged from personal or external life circumstances (6%) to uncertainty about using the referral (6%). Finally, 16% reported no longer needing the guide because their situation had improved.

## DISCUSSION

This study demonstrated that implementing an ED-based FI screening and referral program is feasible and can reduce FI levels among patients. Our findings also revealed significant demographic disparities in FI rates in the ED. Although the intervention was beneficial, certain groups benefited more than others, and several barriers hindered the referral’s optimal utilization.

Compared to other ED-based interventions focusing on FI, which often involve smaller samples or rely solely on a single screener,^2^,^8^,^9^ this study offers several notable strengths. First, it employed multiple validated measures (the Hunger Vital Sign and the 10-item USDA Adult Food Security Survey Module) to screen 6,339 patients—a relatively large sample size for this setting—and assess varying degrees of FI severity. Second, its longitudinal design with three- and six-week follow-up enabled a better understanding of how FI status evolved over time post-intervention, rather than relying on a single snapshot. Third, qualitative insights on patients’ barriers provided a more nuanced perspective of real-world challenges faced by individuals at risk. These features helped to contextualize the findings while indicating where further refinement of ED-based interventions may be needed.

### Demographic Disparities in Food Insecurity

Our ED’s overall FI rate of 16.9% highlights the substantial presence of FI in vulnerable populations, even in affluent areas like Orange County, CA, which had a general population FI rate of 10.4% in 2022.^3^ This elevated rate in the ED aligns with studies that have found higher FI rates in ED settings compared to the general population, suggesting a correlation between FI and ED visits.^2^, ^15^ Analysis revealed significant disparities, with Black non-Hispanic individuals experiencing the highest FI rates (24.7%). This mirrors literature indicating that systemic inequalities, such as historical marginalization, limited access to economic opportunities, and healthcare disparities, contribute to higher FI rates among racial and ethnic minorities.^16^, ^17^ Addressing these underlying social determinants is crucial for developing effective interventions.

Spanish speakers exhibited a significantly higher FI rate of 28.7% compared to 15.9% among English speakers (*P* < 0.001). This underscores the impact of language barriers in regular access to food resources. Implementing multilingual outreach programs and providing resources that are linguistically and culturally tailored could enhance awareness and utilization among non-English-speaking populations.^18^ Age-related differences in FI were also evident, with the highest prevalence among individuals 45–59 years of age (22.6%) and the lowest among those ≥60 (11.3%). The higher rates in middle-aged groups may be related to economic pressures, employment instability, and health issues that impact their ability to secure adequate nutrition.^19^ For seniors, increased social support (eg, such as through increased asset limits for Supplemental Nutrition Assistance Program eligibility) likely contributed to their relatively lower rates of FI.^20^

### Effectiveness of the Food Resource Guide Intervention

The implementation of the food resource referral intervention demonstrated a significant reduction in FI scores among participants. Participants saw their mean FI score decrease from 6.67 to 4.75 within three weeks, which stabilized at 4.25 during the six-week follow-up. While the initial drop from enrollment to both follow-ups was statistically significant, the slight decrease from 4.75 to 4.25 between the three- and six-week follow-ups was not significant. Notably, RAs did not actively advocate for referral usage during follow-up calls. Enhancing follow-up interventions by incorporating personalized support, such as assistance with resource navigation or scheduling visits to food pantries, could further improve outcomes. Previous studies have shown that active follow-up and collaboration by community health workers can lead to significant improvements in social determinants of health.^21^, ^22^ Particularly, *promotoras de salud* (community health workers) in Hispanic communities have been effective in building trust and facilitating access to resources to overcome health barriers.^23^, ^24^ Implementing similar strategies may increase the effectiveness of FI interventions in the ED setting.

Although the unadjusted analysis indicated that participants who used the paper referral guide experienced greater reductions in FI scores than non-users (*P* = 0.03), the multiple linear regression analysis revealed that this difference was not statistically significant after accounting for baseline differences, age, socioeconomic status, and other relevant factors (β −0.649, SE 0.645, t −1.006, *P* = 0.316). This suggests that the paper referral guide, on its own, may not have been the primary driver behind the reduction in FI scores. However, the regression analysis showed that participants with higher baseline FI scores were more likely to experience greater reductions in FI (β −0.655, SE 0.095, t −6.907, *P* < 0.001), indicating that individuals with higher levels of FI at enrollment tended to benefit the most from the intervention. Overall, the improvements in FI scores appear to be primarily explained by participants’ higher initial FI levels rather than by guide use alone, suggesting that individuals with the greatest need for food resources experienced the most substantial improvements from the intervention.[Table t3-wjem-26-396]

The multiple linear regression analysis revealed that ethnicity also played an important role in FI score reductions, with Hispanic or Latino/a participants experiencing significantly greater improvements compared to White non-Hispanic participants (β −1.284, SE 0.615, t −2.087, *P* = 0.039). This suggests that Hispanic or Latino/a individuals may have responded particularly well to the intervention or that other unmeasured factors, such as differences in community support or resource utilization, might have influenced these outcomes.

### Barriers to Use of Food Assistance Resources

Of the 163 three-week follow-ups completed, only 35 participants cited specifically using the paper food resource guide since leaving the ED. Thus, understanding the barriers to using those assistance resources is crucial. The most common barrier identified among patients who didn’t use the paper guide was time constraints (20.31%). These time constraints could be related to an intersection of socioeconomic constraints, such as having multiple jobs and family responsibilities.^25^, ^26^ Additionally cited barriers reflect other social determinants of health, such as transportation issues (16.41%), which have been shown to correlate with FI.^27^ Providing transportation support or partnering with local organizations to deliver food could minimize these barriers. Programs that offer tailored home delivery services such as grocery bags or the Meals on Wheels program for seniors have been shown to increase access to food assistance for those with similar barriers.^28^–^30^ Many outpatient clinics have demonstrated success in reducing FI through food prescription programs.^31^,^32^ Implementing hospital-based prescriptive food services could help bridge some of the gaps observed in this study.^33^

A notable portion of guide non-users cited losing the referral or not recalling receiving it in the first place (12.5%), pointing to a need for more reliable delivery of food assistance information. These challenges highlight the necessity for a more systematic and technology-driven approach in delivering food resources to patients with FI. Given that this study is among several that have successfully screened ED patients for FI using the Hunger Vital Sign,^2^, ^34^ future strategies should consider incorporating universal food security screening during ED intake, using electronic health records (EHR) to streamline the process. Some prominent EHR systems, such as Epic’s Foundation System (Epic Systems Corporation, Verona WI) have already integrated the Hunger Vital Sign screener,^35^ making it feasible to conveniently assess all patients. Patients identified as food insecure through universal screening could then be automatically flagged to receive resource referral guides included in their discharge papers, or even through scheduled follow-up texts and/or emails. This approach would standardize the screening and intervention process, reducing the chance of human error (such as patients not receiving the guide) and potentially increasing the use of available food resources.

## LIMITATIONS

This study has several limitations that should be considered when interpreting the results. First, as a single-center observational study, the findings may not be generalizable to other settings or populations. The study was conducted at one urban, academic ED, and the sample may not represent the broader population of ED patients or those in different geographic areas. Another notable limitation is the lack of a formal control group. Because participants who received the food resource referral were not compared directly against a similar group without the intervention, it remains uncertain whether the observed reductions in FI scores can be fully attributed to the referral program. External unmeasured factors, such as shifts in employment or economic fluctuations, may also have influenced reductions in FI. As a result, we cannot definitively conclude that the referral alone caused the decrease in FI scores.

Additionally, attrition throughout the study was high, which may affect both the magnitude and direction of FI score reductions. Although we targeted at least 95 participants for the USDA Adult Food Security Survey Module, only 161 (25.6%) of the 630 enrolled patients completed the three-week follow-up phone calls, and 48 (7.6%) completed the six-week follow-up. This low follow-up rate not only reduces the overall sample size but also introduces the possibility of attrition bias: it is possible that those who continued in the study either experienced greater improvements (making our observed results more optimistic) or were, conversely, more motivated to respond due to persistent challenges (leading to an underestimation of potential improvements). Although baseline demographics between respondents and non-respondents were compared to partially assess non-response bias, the inability to perform wave analysis and additional follow-up assessments limited our capacity to fully evaluate how attrition might have skewed the results.

The reliance on verbal administration of the screening questions may have affected the accuracy of the responses. Previous literature suggests that written questionnaires during administration of the Hunger Vital Sign may yield more accurate responses due to increased patient comfort and privacy.^36^ This could have influenced the identification of food-insecure individuals. Screening was also limited by language barriers. Due to limited availability of bilingual RAs, Spanish speakers were under-screened, and patients who spoke other languages were rarely screened due to their exclusion from full enrollment. In an area as linguistically diverse as Orange County,^37^ this limits the generalizability of certain results and suggests that language-inclusive strategies are necessary for comprehensive screening. This is reflective of the overall limitation of the study’s reliance on convenience sampling, which is less generalizable compared to random sampling or universal screening.

Finally, assessment of the food resource intervention was held back by limitations. The follow-up questions were adapted from the USDA Adult Food Security Survey Module to assess food security over the previous three weeks. However, the module is generally validated for a 12-month period.^38^ This adaptation may have affected the validity of the results. It should be noted, too, that the follow-up periods of three and six weeks were relatively short compared to the persistent nature of FI.^39^

## CONCLUSION

This study reaffirmed the persistent issue of food insecurity among ED patients, particularly within vulnerable demographics. The implementation of an ED-based food resource referral guide was associated with a significant decrease in FI scores, demonstrating its potential effectiveness as an intervention. However, barriers such as time constraints and transportation issues emphasize the need for more personalized and systematic support systems. Future strategies could incorporate universal food security screening during ED intake and offer personalized follow-up interventions to address these nuanced barriers and improve outcomes for food-insecure individuals.

## Supplementary Information





## Figures and Tables

**Figure 1 f1-wjem-26-396:**
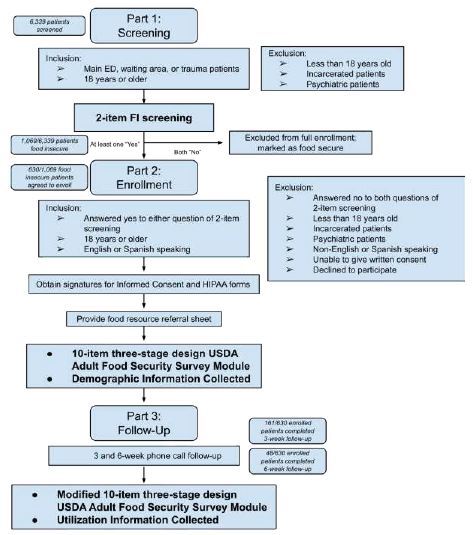
Flowchart depicting screening, enrollment, and follow-up methodology. *HIPAA*, Health Insurance Portability and Accountability Act*; USDA*, US Department of Agriculture.

**Table 1 t1-wjem-26-396:** Food insecurity prevalence by demographics.

Category	n	n Positive	n Negative	Rate FI Positive (%)
All	6,339	1,069	5,270	16.9%
Sex				
Men	3,259	584	2,675	17.9%
Women	3,080	485	2,595	15.8%
Race				13.8%
White, non-Hispanic	2,595	358	2,237	13.8%
Black, non-Hispanic	271	67	204	24.7%
Hispanic or Latino/a	2,390	515	1,875	21.6%
Asian or Pacific Islander	828	75	753	9.1%
American Indian, Alaskan, or Hawaiian Native	60	14	46	23.3%
Other	195	40	155	20.5%
Language				
English	5,622	896	4,726	15.9
Spanish	534	153	381	28.7%
Other	183	20	163	12.3%
Age				
18–29	1,287	211	1,076	16.4%
30–44	1,442	293	1,149	20.3%
45–59	1,390	314	1,076	22.6%
≥60	2,220	251	1,969	11.3%

*FI*, food insecurity.

**Table 2:** Multiple linear regression analysis predicting change in food insecurity scores.

Predictor	Estimate (β)	Std. Error	t value	P-value
(Intercept)	3.1162	1.4927	2.0876	0.04
Guide use (Guide users)	−0.5046	0.6791	−0.7430	0.46
Initial FI Score upon enrollment	−0.6520	0.0972	−6.7087	<0.01
Age	0.0006	0.0004	1.4562	0.15
Gender				
Women	0.9453	0.5353	1.7661	0.08
Ethnicity				
Black, non-Hispanic	−21071	1.1498	−1.8326	0.07
Hispanic or Latino/a	−1.4533	0.6428	−2.2608	0.03
Asian or Pacific Islander	−2.0153	1.2529	−1.6085	0.11
Other	−0.8435	1.2998	−0.6490	0.52
Language				
Spanish	0.7896	1.0656	0.7410	0.46
Other	−0.4686	2.5972	−0.1804	0.86
Marital Status				
Married	−0.1308	0.6784	−0.1928	0.85
Domestic partnership	−3.8145	3.2871	−1.1605	0.25
Education Level				
Elementary/high school/ GED	−0.0109	1.1640	−0.0094	>0.99
Some college	−0.4453	1.2090	−0.3683	0.71
2-year college (AA/AS)	−1.4074	1.4004	−1.0050	0.32
4-year college degree (BA/BS)	−1.7590	1.4477	−1.2151	0.23
Master’s degree	0.2879	2.5746	0.1118	0.91

*FI*, food insecurity; *GED*, General Educational Development.

**Table 3 t3-wjem-26-396:** Barriers to use of food assistance resources.

Theme code	Example quote from notes taken during phone follow-up conversation	% of Patient interviews including theme (N=128)
Time Constraints	“Haven’t had the time yet” “Busy with work” “Busy with school and other things” “Homeless, so has not had time to go”	20.31% (n=26)
Transportation Issues	“Homeless, so no transportation” “Don’t have a car” “Transportation Issues”	16.41% (n= 21)
Not Needed Anymore	“I do not need it anymore” “Using EBT at the moment but will still use food pantries in the future” “Already have it covered by psychiatric coverage”	15.63% (n=20)
Health Issues/Medical Recovery	“Sick often” “Been recovering from treatment, sleeping a lot, feels weak” “Admitted to the hospital again and did not feel healthy enough to go out.” “In a rehabilitation facility at the moment”	14.06% (n=18)
Didn’t Receive or Lost Referral	“Claims did not receive the food pantry listing, wants us to mail” “Claims did not receive the paper” “Did not know about referral”	12.5% (n=16)
Forgot About Referral	“Forgot about it” “Forgot it at the hospital” “Forgot about it because of medication”	8.59% (n=11)
External Life Circumstances	“Staying in a shelter” “Just became homeless/evicted” “Not mobile but has the printout.”	6.25% (n=8)
Uncertainty About Use	“Said she got a job, may or may not use the service, we can call her back but she sounded like she might not really use it” “May or may not use it” “Still unsure about using the referral”	6.25% (n=8)

*EBT*, Electronic Benefits Transfer.
