# Supplementary material for: Feasibility of an Emergency Department-based Food Insecurity Screening and Referral Program
Source: West J Emerg Med. 2025 Mar 15;26(3):396–405. doi: 10.5811/westjem.40006 (PMC12208039; doi:10.5811/westjem.40006)
Supplement: Supplementary file 1 [file wjem-26-396-SupplementaryA.pdf]

Food Security Survey (Screening)

Screened ID	__________________________
Age	__________________________
Gender	◯ Male<br>◯ Female
Preferred Language	◯ English<br>◯ Spanish<br>◯ Vietnamese<br>◯ Other (please specify)
Other Language	__________________________
Ethnicity	◯ White, non-Hispanic<br>◯ Black, non-Hispanic<br>◯ Hispanic or Latino/a<br>◯ Asian or Pacific Islander<br>◯ American Indian, Alaskan or Hawaiian Native<br>◯ Other (please specify)
Other Ethnicity	__________________________
Within the past 12 months, I worried whether my food would run out before I got money to buy more.	◯ Yes<br>◯ No
Within the past 12 months, the food I bought just didn't last and I didn't have money to get more.	◯ Yes<br>◯ No
This patient is considered FOOD INSECURE! Please approach/consent this patient for the second part of the study. Also, please remember to give the referral regardless of whether or not they complete the second portion. If they finish the second portion, they will also receive a compensatory $5 gift card.<br><br>Did you enroll?	◯ Yes<br>◯ No
Why not?	__________________________

Part 2: Data Abstraction and FI Survey

Study ID	______________________
Date	______________________
MRN	______________________
For Research Associate: Chief Complaint	______________________
For Research Associate: Emergency Serverity Index	______________________
Name	______________________<br>((Last,First))
Education Level	◯ No schooling completed<br>◯ Elementary/High School GED<br>◯ Some College<br>◯ 2-Year College (AA/AS)<br>◯ 4-Year College Degree (BA/BS)<br>◯ Master's Degree<br>◯ Doctoral Degree (PhD, PsyD)<br>◯ Professional Degree (MD,JD)
Marital Status	◯ Single<br>◯ Married<br>◯ Domestic Partnership
Besides yourself, how many adults live in the household?	______________________
How many children do you have in your household?	______________________
Please list the ages of your children	______________________
My Emergency Department visit today is related to my food insecurity status	◯ 1- Strongly disagree<br>◯ 2- Disagree<br>◯ 3- Neither agree or disagree<br>◯ 4- Agree<br>◯ 5- Strongly agree
Best Means of Contact	☐ Call<br>☐ Text<br>☐ Email
Phone Number	______________________<br>(Numbers ONLY (no dash or parenthesis))

Service Provider	__________________________________
Best Time of Contact	◯ Morning<br>◯ Lunch<br>◯ Afternoon<br>◯ Evening
Email Address (for compensation)	__________________________________
Other Contact	__________________________________
"In the past 12 months, I was worried whether my food would run out before I got money to buy more."	◯ Often true<br>◯ Sometimes true<br>◯ Never true<br>◯ Don't Know
"In the past 12 months, the food that I bought just didn't last, and I didn't have money to get more."	◯ Often true<br>◯ Sometimes true<br>◯ Never true<br>◯ Don't Know
"In the past 12 months, I couldn't afford to eat balanced meals."	◯ Often true<br>◯ Sometimes true<br>◯ Never true<br>◯ Don't Know
In the last 12 months, did you or other adults in the household ever cut the size of your meals or skip meals because there wasn't enough money for food?	◯ Yes<br>◯ No<br>◯ Don't Know
How often did this happen?	◯ Almost every month<br>◯ Some months, but not every month<br>◯ Only 1 or 2 months<br>◯ Don't Know
In the last 12 months, did you ever eat less than you felt you should because there wasn't enough money for food?	◯ Yes<br>◯ No<br>◯ Don't Know
In the last 12 months, were you ever hungry but didn't eat because there wasn't enough money for food?	◯ Yes<br>◯ No<br>◯ Don't Know
In the last 12 months, did you lose weight because there wasn't enough money for food?	◯ Yes<br>◯ No<br>◯ Don't Know
In the last 12 months, did you or other adults in your household ever not eat for a whole day because there wasn't enough money for food?	◯ Yes<br>◯ No<br>◯ Don't Know

If yes, how often did this happen?	◯ Almost every month<br>◯ Some months, but not every month<br>◯ Only 1 or 2 months<br>◯ Don't Know
Thank you for taking the time to answer these questions. Please call us back at [REDACTED FOR SUBMISSION]<br>if you have any questions about your participation in this study!<br><br>As a reminder, we will be calling you in 3 more weeks to complete a brief survey. Thank you for your time!<br><br>Have you received your referral and gift card?	◯ Yes<br>◯ No
FI Value	________________________________

Liaison: Followup Week 3

Date	______________________
Did you use your food pantry referral since your discharge from the ED?	◯ Yes<br>◯ No
What prevented you from using the food pantry referral?	______________________
How many times have you used the food pantry since your ED visit?	______________________
Which locations have you visited?	______________________
On a scale of 1 to 5, how helpful was this food pantry referral in reducing your concerns about food availability?	◯ 5- Extremely helpful<br>◯ 4- Very helpful<br>◯ 3- Somewhat helpful<br>◯ 2- Slightly helpful<br>◯ 1- Not at all helpful
"In the past 3 weeks, I worried whether our food would run out before I got money to buy more."	◯ Often true<br>◯ Sometimes true<br>◯ Never true<br>◯ Don't Know
"In the past 3 weeks, the food that I bought just didn't last, and I didn't have money to get more."	◯ Often true<br>◯ Sometimes true<br>◯ Never true<br>◯ Don't Know
"In the past 3 weeks, I couldn't afford to eat balanced meals."	◯ Often true<br>◯ Sometimes true<br>◯ Never true<br>◯ Don't Know
In the last 3 weeks, did you ever cut the size of your meals or skip meals because there wasn't enough money for food?	◯ Yes<br>◯ No<br>◯ Don't Know
How often did this happen?	◯ Almost every month<br>◯ Some months, but not every month<br>◯ Only 1 or 2 months<br>◯ Don't Know
In the last 3 weeks, did you ever eat less than you felt you should because there wasn't enough money for food?	◯ Yes<br>◯ No<br>◯ Don't Know
In the last 3 weeks, were you every hungry but didn't eat because there wasn't enough money for food?	◯ Yes<br>◯ No<br>◯ Don't Know

In the last 3 weeks, did you lose weight because there wasn't enough money for food?	◯ Yes<br>◯ No<br>◯ Don't Know
In the last 3 weeks, did you or other adults in your household ever not eat for a whole day because there wasn't enough money for food?	◯ Yes<br>◯ No<br>◯ Don't Know
If yes, how often did this happen?	◯ Almost every month<br>◯ Some months, but not every month<br>◯ Only 1 or 2 months<br>◯ Don't Know
Comments	__________________________________
Thank you for taking the time to answer these questions. Please call us back at [REDACTED FOR SUBMISSION] if you have any questions about your participation in this study.<br><br>At 3-week followup: As a reminder, we will be calling you again in 3 more weeks to complete a brief survey. Thank you for your time."<br><br>Did you tell them this?	◯ Yes<br>◯ No
FI Value	__________________________________

Liaison: Followup Week 6

Date	__________________________________
Did you use your food pantry referral since your discharge from the ED?	◯ Yes<br>◯ No
What prevented you from using the food pantry referral?	__________________________________
How many times have you used the food pantry since your ED visit?	__________________________________
Which locations have you visited?	__________________________________
On a scale of 1 to 5, how helpful was this food pantry referral in reducing your concerns about food availability?	◯ 5- Extremely helpful<br>◯ 4- Very helpful<br>◯ 3- Somewhat helpful<br>◯ 2- Slightly helpful<br>◯ 1- Not at all helpful
"In the past 3 weeks, I worried whether our food would run out before I got money to buy more."	◯ Often true<br>◯ Sometimes true<br>◯ Never true<br>◯ Don't Know
"In the past 3 weeks, the food that I bought just didn't last, and I didn't have money to get more."	◯ Often true<br>◯ Sometimes true<br>◯ Never true<br>◯ Don't Know
"In the past 3 weeks, I couldn't afford to eat balanced meals."	◯ Often true<br>◯ Sometimes true<br>◯ Never true<br>◯ Don't Know
In the last 3 weeks, did you ever cut the size of your meals or skip meals because there wasn't enough money for food?	◯ Yes<br>◯ No<br>◯ Don't Know
How often did this happen?	◯ Almost every month<br>◯ Some months, but not every month<br>◯ Only 1 or 2 months<br>◯ Don't Know
In the last 3 weeks, did you ever eat less than you felt you should because there wasn't enough money for food?	◯ Yes<br>◯ No<br>◯ Don't Know
In the last 3 weeks, were you every hungry but didn't eat because there wasn't enough money for food?	◯ Yes<br>◯ No<br>◯ Don't Know

In the last 3 weeks, did you lose weight because there wasn't enough money for food?	◯ Yes<br>◯ No<br>◯ Don't Know
In the last 3 weeks, did you or other adults in your household ever not eat for a whole day because there wasn't enough money for food?	◯ Yes<br>◯ No<br>◯ Don't Know
If yes, how often did this happen?	◯ Almost every month<br>◯ Some months, but not every month<br>◯ Only 1 or 2 months<br>◯ Don't Know
Comments	__________________________________
Thank you for taking the time to answer these questions. Please call us back at [REDACTED FOR SUBMISSION] if you have any questions about your participation in this study.<br><br>At 3-week followup: As a reminder, we will be calling you again in 3 more weeks to complete a brief survey. Thank you for your time."<br><br>Did you tell them this?	◯ Yes<br>◯ No
FI Value	__________________________________
