# Supplementary material for: Feasibility of an Emergency Department-based Food Insecurity Screening and Referral Program
Source: West J Emerg Med. 2025 Mar 15;26(3):396–405. doi: 10.5811/westjem.40006 (PMC12208039; doi:10.5811/westjem.40006)
Supplement: Supplementary file 2 [file wjem-26-396-supplememtaryB.pdf]

**UCI HEALTH**

# ORANGE COUNTY

Food Assistance and Referrals

## FOOD PANTRIES:

### FEEDOC.ORG

Are you or your family finding it hard to afford enough food? You're not alone, and there's help nearby. Second Harvest Food Bank of Orange County offers various ways to get free, nutritious food easily. Here's how you can get the help you need:

1. **Visit feedoc.org and click on the "Find Food" section.**
2. **Click on "Click HERE for a current list of Second Harvest pantries and partners."**
3. **Search the list for a food pantry near you.**

**SCAN HERE to visit feedoc.org**

### OCFOODHELP.ORG FOOD MAP

Searching for nearby food help? Use OC Food Help's simple interactive map. Just enter your location to see nearby food pantries, meal services, and affordable markets. Access immediate, nearby assistance with easy-to-find information on operating hours and contacts.
Start your search on ocfoodhelp.org/orange-county-free-food-map or scan below.

**SCAN HERE to visit ocfoodhelp.org's food map.**

## ADDITIONAL RESOURCES

### NEED MORE ASSISTANCE? CONTACT 211OC.

For a comprehensive directory of services in Orange County – from housing and job assistance to health resources – **dial 211** or **visit 211oc.org**. It's a one-stop hub, available 24/7, to connect you with local programs and services to meet your needs.

**SCAN HERE TO ACCESS 211OC's vast resources.**

CalFresh FOOD

### What is CalFresh?

CalFresh is California's food stamps (SNAP) program.
CalFresh provides monthly food assistance to individuals and families with low incomes to ensure they can meet their nutritional needs.

### How does it work?

**1. Apply**
Your county will call you within a week or two for an eligibility interview.

**2. Get money**
If approved, you will get an EBT card for groceries within 10 days.

**3. Buy groceries**
EBT cards work in most food stores and farmers' markets.

### What will I need?

1. A copy of your ID.

2. Proof of any income.

3. Proof of immigration status. (For non-citizens)

**SCAN HERE to apply for CalFresh.**
Alternatively, visit **getcalfresh.org** or **call 211**.

UCI HEALTH

# ORANGE COUNTY

Food Assistance and Referrals

## ORANGE

**First Presbyterian Church of Orange**
191 N Orange St, Orange 92866
Wednesday 10:00 AM-12:00 PM
Sunday 2:00 PM-3:30 PM

**The Hub Resource Center ☆**
517 W Struck Ave, Orange 92867
Monday-Friday 9:00 AM-3:00 PM

**St. Norbert's Christan Service**
300 East Taft Ave, Orange 92865
Saturday 10:30 AM-12:00 PM (Drive-through)

**La Purisima Church/Trinity Guild**
11712 N. Hewes, Orange 92869
Monday, Wednesday, Friday 10:00-12:00 PM
Saturday 8:30-10:30 AM

## SANTA ANA

**Catholic Charities of Orange County**
2020 W. Chestnut Ave, Santa Ana 92703
Tuesday-Thursday 7:30 AM-12:00 PM

**Delhi Center ☆**
505 E. Central Ave, Santa Ana 92707
Monday-Thursday 8:30 AM- 7:00 PM
Friday-Saturday 8:30 AM-4:00 PM

**Mercado El Sol**
**(Application & appointment required)**
1002 N Broadway, Santa Ana, CA 92701
Monday, Tuesday, Thursday 12:00-2:00 PM
Wednesday 1:00-2:00 PM
Friday 3:00-5:00 PM

## GARDEN GROVE

**Christ Cathedral Community Outreach**
12141 S. Lewis St., Garden Grove 92840
Thursday 10:00AM-2:00PM

**Helping Others Prepare for Eternity (H.O.P.E.)**
11022 Acacia Pkwy #C, Garden Grove 92840
Monday-Friday 1:00 PM-3:30 PM

**Islamic Society of OC**
9752 13th Street, Garden Grove 92844
Monday 10:00 AM-12:00 PM

**St. Anselm Episcopal Church**
13091 Galway St., Garden Grove 92844
Saturday 11:00AM-12:00 PM

## COSTA MESA

**Share Our Selves ☆**
1550 Superior Ave., Costa Mesa 92627
Monday-Friday 8:00 AM-4:30 PM

## ANAHEIM

**Gilbert High- El Mercadito PSP**
1800 W Ball Road, Anaheim 92804
Tuesday 1:30 PM-3:00PM
Thursday 8:00 AM-10:00 AM

**Salvation Army ☆**
1515 W. North Street, Anaheim 92801
Tuesday & Thursday 1:00-2:30 PM

**St. Boniface Catholic Church SVDP**
124 N. Resh St, Anaheim 92805
Monday, Wednesday, & Friday 9:00-11:00 AM

## FULLERTON

**Pathways of Hope**
611 S. Ford Ave, Fullerton 92832
Monday-Friday 1:00-4:00 PM

**Seventh-day Adventist Church Fullerton**
2355 West Valencia Drive, Fullerton 92833
Tuesday 8:30 AM-11:30 AM

## WESTMINSTER

**The Men with Vision Food Pantry ☆**
13552 Goldenwest Street, Westminster 92683
Thursday 2:00-4:00 PM

**His Place Church**
14061 Chestnut Street, Westminster 92683
Friday, 2nd/ 4th of the month 8:00 AM-10:00 AM

## IRVINE

**Families Forward**
8 Thomas, Irvine 92618
Monday-Thursday 10:00-4:00 PM
(Note: For first time visitors, must register at front desk and provide proof of OC residency)

**Mariner's Church Community Center ☆**
5001 Newport Coast Drive, Irvine 92603
Tuesday-Friday 9:00 AM-1:00 PM

**South County Outreach**
7 Whatney, Suite B, Irvine 92618
Monday-Thursday 9:00-2:00 PM
(Note: Requires application: https://tinyurl.com/ym95862w)

## HOT MEALS

**Someone Cares Soup Kitchen ☆**
720 W 19th St, Costa Mesa, CA 92627
Monday-Saturday 12:00-3:00 PM
Sunday 9:00-11:00 AM

**Orangethorpe Christian Church - Hot Meals Ministry**
2200 W. Orangethorpe Ave., Fullerton 92833
Monday 6:00-7:00 PM

**Mary's Kitchen ☆**
790 E Debra Ln, Anaheim, CA 92805
Monday-Saturday 9:00-3:00 PM

UCI HEALTH

# CONDADO DE ORANGE

Asistencia Alimentaria y Referencias

## DESPENSAS DE ALIMENTOS:

### FEEDOC.ORG

¿Usted o su familia tienen dificultades para costear suficiente comida? No están solos y hay ayuda cerca. El Banco de Alimentos Second Harvest del Condado de Orange ofrece diversas formas de obtener comida nutritiva y gratuita con facilidad. Aquí le explicamos cómo puede obtener la ayuda que necesita:

1. **Visite feedoc.org y haga clic en la sección "Necesito Comida".**
2. **Haga clic en "Haga clic AQUÍ para obtener una lista actualizada de despensas y socios de Second Harvest".**
3. **Busque en la lista una despensa de alimentos cerca de usted.**

**ESCANEE AQUÍ para visitar feedoc.org**

### OCFOODHELP.ORG MAPA DE ALIMENTOS

¿Buscando ayuda alimentaria cercana? Utilice el mapa interactivo simple de OC Food Help. Solo ingrese su ubicación para ver despensas de alimentos, servicios de comidas y mercados asequibles cercanos. Acceda a asistencia inmediata y cercana con información fácil de encontrar sobre horarios de operación y contactos.
Inicie su búsqueda en ocfoodhelp.org/orange-county-free-food-map o escanee abajo.

**ESCANEE AQUÍ para visitar el mapa de alimentos de ocfoodhelp.org.**

## RECURSOS ADICIONALES

### ¿NECESITA MÁS ASISTENCIA? CONTACTE A 211OC.

Para obtener un directorio completo de servicios en el Condado de Orange – desde asistencia para vivienda y empleo hasta recursos de salud – **marque al 211** o **visite 211oc.org**. Es un centro integral, disponible 24/7, para conectarlo con programas y servicios locales que satisfagan sus necesidades.

**ESCANEE AQUÍ PARA ACCEDER a los amplios recursos de 211OC.**

---

CalFresh FOOD

### ¿Qué es CalFresh?

CalFresh es el programa de cupones para alimentos (SNAP) de California. CalFresh proporciona asistencia alimentaria mensual a individuos y familias con bajos ingresos para asegurar que puedan satisfacer sus necesidades nutricionales.

### ¿Cómo funciona?

**1. Solicitar**
Su condado le llamará dentro de una o dos semanas para una entrevista de elegibilidad.

**2. Recibir dinero**
Si es aprobado, recibirá una tarjeta EBT para comestibles dentro de 10 días.

**3.Comprar comestibles**
Las tarjetas EBT funcionan en la mayoría de las tiendas de alimentos y mercados de agricultores.

### ¿Qué necesitaré?

1. Una copia de su identificación.

2. Prueba de cualquier ingreso.

3. Prueba de estatus migratorio. (Para no ciudadanos)

PASS

**ESCANEE AQUÍ para solicitar CalFresh.**
Alternativamente, visite **getcalfresh.org** o **llame al 211**.

## UCI HEALTH

# CONDADO DE ORANGE

Asistencia Alimentaria y Referencias

## ORANGE

**First Presbyterian Church of Orange**
191 N Orange St, Orange 92866
Miércoles 10:00 AM-12:00 PM
Domingo 2:00 PM-3:30 PM

**The Hub Resource Center** ☆
517 W Struck Ave, Orange 92867
Lunes-Viernes 9:00 AM-3:00 PM

**St. Norbert's Christan Service**
300 East Taft Ave, Orange 92865
Sábado 10:30 AM-12:00 PM (Auto-servicio)

**La Purisima Church/Trinity Guild**
11712 N. Hewes, Orange 92869
Lunes, Miércoles, Viernes 10:00-12:00 PM
Sábado 8:30-10:30 AM

## SANTA ANA

**Catholic Charities of Orange County**
2020 W. Chestnut Ave, Santa Ana 92703
Martes-Jueves 7:30 AM-12:00 PM

**Delhi Center** ☆
505 E. Central Ave, Santa Ana 92707
Lunes-Jueves 8:30 AM- 7:00 PM
Viernes-Sábado 8:30 AM-4:00 PM

**Mercado El Sol**
**(Se requiere solicitud y cita previa)**
1002 N Broadway, Santa Ana, CA 92701
Lunes, Martes, Jueves 12:00-2:00 PM
Miércoles 1:00-2:00 PM
Viernes 3:00-5:00 PM

## GARDEN GROVE

**Christ Cathedral Community Outreach**
12141 S. Lewis St., Garden Grove 92840
Jueves 10:00AM-2:00PM

**Helping Others Prepare for Eternity (H.O.P.E.)**
11022 Acacia Pkwy #C, Garden Grove 92840
Lunes-Viernes 1:00 PM-3:30 PM

**Islamic Society of OC**
9752 13th Street, Garden Grove 92844
Lunes 10:00 AM-12:00 PM

**St. Anselm Episcopal Church**
13091 Galway St., Garden Grove 92844
Sábado 11:00AM-12:00 PM

## COSTA MESA

**Share Our Selves** ☆
1550 Superior Ave., Costa Mesa 92627
Lunes-Viernes 8:00 AM-4:30 PM

## ANAHEIM

**Gilbert High- El Mercadito PSP**
1800 W Ball Road, Anaheim 92804
Martes 1:30 PM-3:00PM
Jueves 8:00 AM-10:00 AM

**Salvation Army** ☆
1515 W. North Street, Anaheim 92801
Martes & Jueves 1:00-2:30 PM

**St. Boniface Catholic Church SVDP**
124 N. Resh St, Anaheim 92805
Lunes, Miércoles, & Viernes 9:00-11:00 AM

## FULLERTON

**Pathways of Hope**
611 S. Ford Ave, Fullerton 92832
Lunes-Viernes 1:00-4:00 PM

**Seventh-day Adventist Church Fullerton**
2355 West Valencia Drive, Fullerton 92833
Martes 8:30 AM-11:30 AM

## WESTMINSTER

**The Men with Vision Food Pantry** ☆
13552 Goldenwest Street, Westminster 92683
Jueves 2:00-4:00 PM

**His Place Church**
14061 Chestnut Street, Westminster 92683
Viernes, 2.°/4.° del mes 8:00 AM-10:00 AM

## IRVINE

**Families Forward**
8 Thomas, Irvine 92618
Lunes-Jueves 10:00-4:00 PM
(Nota: Para los visitantes por primera vez, deben registrarse en la recepción y proporcionar prueba de residencia en OC.)

**Mariner's Church Community Center** ☆
5001 Newport Coast Drive, Irvine 92603
Martes-Viernes 9:00 AM-1:00 PM

**South County Outreach**
7 Whatney, Suite B, Irvine 92618
Lunes-Jueves 9:00-2:00 PM
(Nota: se require solicitud: https://tinyurl.com/ym95862w)

## COMIDAS CALIENTES

**Someone Cares Soup Kitchen** ☆
720 W 19th St, Costa Mesa, CA 92627
Lunes-Sábado 12:00-3:00 PM
Domingo 9:00-11:00 AM

**Orangethorpe Christian Church - Hot Meals Ministry**
2200 W. Orangethorpe Ave., Fullerton 92833
Lunes 6:00-7:00 PM

**Mary's Kitchen** ☆
790 E Debra Ln, Anaheim, CA 92805
Lunes-Sábado 9:00-3:00 PM
